# Diagnostic error in the Emergency Department: follow up of patients with minor trauma in the outpatient clinic

**DOI:** 10.1186/s13049-017-0361-5

**Published:** 2017-02-14

**Authors:** Pieter-Jan Moonen, Luc Mercelina, Willem Boer, Tom Fret

**Affiliations:** 1 Department of Anesthesiology, Critical and Emergency Medicine and Pain Therapy, Ziekenhuis Oost Limburg Genk, Schiepsebos 11, 3600 Genk, Belgium; 2 Department of General Surgery, Ziekenhuis Oost Limburg Genk, Schiepsebos 11, 3600 Genk, Belgium; 3 ᅟ, Ieperstraat 43, 2300 Turnhout, Belgium

**Keywords:** Diagnostic error, Primary missed diagnosis, Minor trauma, Emergency department, Malpractice, Fracture

## Abstract

**Background:**

The Emergency Department (ED) is prone to diagnostic error. Most frequent diagnostic errors involved “minor” trauma. Our goal was to determine how frequently a missed diagnosis was detected during follow up and to determine the frequency and causes of primary missed diagnosis and diagnostic error.

**Methods:**

A retrospective single centre study review, during 6 months including all patients presenting to the outpatient clinic after ED admission with a minor trauma. We defined primary missed diagnosis versus diagnostic error. Demographic data were collected in Excel file and analyzed using Χ^2^ and unpaired T-test.

**Results:**

Inclusion of 56 patients leading to 57 missed diagnoses representing 1.39% of all minor trauma patients presenting to the ED. History and physical examination notes were incomplete or inadequate in respectively 17/56 and 20/56. Most frequently missed diagnoses were ankle (13/57), wrist (8/57) and foot (7/57) fractures. Causes for diagnostic error could be categorized into two main groups: failure to perform adequate history taking and/or physical examination and failure to order or correctly interpret technical investigation. In 6 cases (0.14%) diagnostic error was confirmed. All other cases were defined as primary missed diagnosis.

**Discussion:**

Emergency physicians have to remain vigilant to prevent and avoid primary missed diagnosis (PMD) and diagnostic error (DE), certainly in case of minor trauma patients, representing a large proportion of ED patients. We observed a prevalence of 1.39% of missed diagnoses within a six month study period. This is comparable to previous studies (1% ). However in our study both primary missed diagnoses and DE were included. Using this definition we saw that only one case could be attributed to negligence and DE had a prevalence of 0.14% (6 cases). X-rays remain the mainstay investigation for minor trauma patients, however in certain selected cases (pelvic and spinal trauma) we advise early CT-scan.Follow up in an outpatient clinic or other forms of planned follow up have to be provided and help to reduce PMD and DE.

**Conclusion:**

Both primary missed diagnosis and diagnostic error have relatively low prevalence but have a serious impact on patients, hospitals and medical services. Planned follow up after adequate explanation can help to prevent diagnostic error and detect primary missed diagnosis, thereby reducing time to final diagnosis and risks for medico legal litigation. Reassessment of diagnostic error on a timely basis can be used as a key performance indicator in a quality assessment program.

## Background

The Emergency Department (ED) is a high pressure, challenging work place. Clinical practice, due to excessive stresses and strains, is prone to diagnostic error (DE) and subsequent litigation [1–4]. Diagnostic errors have implications for patient care and may cause prolonged work incapability, increased (medical) costs [4], give rise to negative publicity and may eventually lead to medico legal prosecution [1, 3].

Several factors contribute to a high pressure working environment: large numbers of patients, crowding, large variety in pathology, unclear acuity of complaints, frequent distractions and shiftwork can all contribute [1, 5, 6].

In the last two decades litigation against both doctors and ED’s relating to DE has increased significantly. Between 4 and 6 percent of all medical malpractice claims in the United States stem from the ED [1, 3]. Thomas et al. [5] demonstrated a relatively low prevalence of adverse events (defaults in medical management) in the ED. However, when an adverse event was present, and the fault was attributed to an ED physician, 94.8% of cases was considered to be negligent. In malpractice claim studies the most frequent cause for litigation was missed diagnosis [1, 3, 5].

Most frequent DE or missed diagnoses in the ED involved “minor” trauma: fractures, luxations and soft tissue lesions [1, 4, 7, 8]. Similarly in pediatric malpractice cases fractures are the most common DE and ED physicians were involved in up to 45% of malpractice lawsuits [7].

The majority of studies concerning missed diagnosis and DE in the ED were performed in Anglo-Saxon countries and there are limited data from European countries. In Belgium no such data have been published.

The goal of our study was to determine how frequently a missed diagnosis was detected during follow up at an outpatient clinic (OPC). We wanted to determine the frequency and causes of primary missed diagnosis and DE.

## Methods

### Definitions

Primary missed diagnosis (PMD): a diagnosis that was unavoidably missed and could only be discovered after follow up and secondary technical investigations (CT/MRI).

Diagnostic error (DE): a diagnosis which, after evaluation of performed clinical and technical investigations, should have been made (in the ED) but was only discovered after follow up [2, 3].

Negligence: care below standard level of performance expected from an average practitioner who treats similar problems at the same time frame [9].

Minor trauma: traumatic or accidental injury with a maximal Injury Severity Score of 9/75, and trauma without necessity for invasive procedures [10].

### Study design and setting

This study was approved by the hospitals ethics committee. It was a single centre study in a regional training hospital in Belgium. The ED has an annual attendance of approximately 44000 patients. An attending staff Radiologist is present at all times and provides a permanent 24 hour service. The ED is staffed by junior and senior residents from different disciplines (surgery, anesthesiology, emergency and internal medicine) under supervision of an Attending Physician. For a 6 month period, 1^st^ of January until the 30^th^ of June 2015, patients medical files were retrospectively reviewed for presence of a new diagnosis in comparison to ED diagnosis.

Patient files were included by doctors working at the OPC. The files were reviewed by a independent investigator for false positive inclusion. After revision of all data the investigator deducted on a case by case basis if a missed diagnosis was a PMD or could be attributed to DE. In case of suspected error the case was passed to a second investigator to confirm or refute DE. In case of confirmed DE the investigators were asked to determine if this error could have been prevented and/or was caused by negligence.

Inclusion criteria: all patients of all ages after ambulatory ED admission, attending a subsequent outpatient follow up clinic and with a different diagnosis in comparison to ED diagnosis.

Exclusion criteria: non-trauma patients, intra-cranial and thoraco-abdominal trauma of internal organs, patients admitted to hospital, loss to follow up, all knee trauma with planned advanced imaging techniques.

Data were collected after research of the patient files and data subsets collected in the ED. Our primary focus concerned human factors (patient factors, cognitive and skillset errors) contributing to diagnostic failure. Due to the retrospective nature we were unable to take systemic factors (pre hospital care, distractors and work environment) in account as factors for diagnostic failure [11].

Collected data from patient files are: hour of ED attendance and time to final diagnosis, age, initial diagnosis, means of transport to ED, presence and adequacy of physical examination and of history, immobilizing therapy in ED, primary technical investigations at the ED and secondary investigations leading to final diagnosis, patient referral, final diagnosis.

Demographic data were analyzed using Χ^2^ and unpaired T-test.

History and physical examination notes were rated on a scale (adequate-incomplete-insufficient) according to adequacy in comparison to teaching gold standards and clinical decision rules [12–14].

## Results

During the 6 month study period 4,025 ambulant patients presented at the ED with minor trauma. One thousand and eight hundred thirty four patients were registered at the OPC within the study period representing an attendance of 45.6%. After patient record review, 56 patients were included, representing 1.39% of all minor trauma patients. Fifty-seven new diagnoses were made. There was a significant statistical difference in age (44 vs. 34, *p* < 0.005) and hour (13.34 h vs. 14.55 h, *p* < 0.035) of presentation of our population in comparison to overall minor trauma patients. The median time between ED presentation and final diagnosis was 9 days.

Most patients presented with direct ankle trauma (11/56), falls (10/56) and traffic accidents (8/56) (Table 1). The majority presented with a unifocal problem (36/56).

**Table 1:** Presenting complaints

Type or area of complaint	Number
Shoulder	2
Elbow	2
Wrist	3
Hand	3
Both arm and leg pain	2
Knee	2
Ankle	11
Foot	5
Leg	3
Spine	2
Chest	1
Nose	2
Traffic accident	10
Accidental fall	8
Total	56

History and physical examination notes were incomplete or inadequate in respectively 17/56 (30.4%) and 20/56 (35.7%). In 10/56 cases physical examination was insufficient in comparison to the noted complaints. In 7/56 cases, both history and physical examination notes were deemed insufficient.

Technical investigations performed at the ED were mostly limited to x-rays. In only 5/56 cases, a CT scan or ultrasound were performed (Table 2). In contrast to the ED the majority of technical investigations ordered in the OPC were CT and MRI scans, cfr. Tables 2 and 3. In 53 cases x-ray protocols were strictly negative, though in three cases a diagnosis was suggested. In all other cases diagnosis was only possible when more advanced techniques were performed.

**Table 2:** Technical investigations in the ED

Tests	Area	Specific location	number
X-ray			
	Face	Nose	1
	Upper limb	Shoulder	7
		Elbow	3
		Wrist	8
		Hand	5
		Finger	2
		Humerus	6
		Radius/ulna	6
	Lower limb	Hip	8
		Knee	9
		Ankle	16
		Foot	9
		Femur	6
		Tibia/fibula	7
	Pelvis		5
	Spinal column	Cervical spine	9
		Thoracic spine	2
		Lumbar spine	4
	Thorax		7
CT scan		Brain	3
		Cervical spine	3
Ultrasound			2
Total			128

**Table 3:** Technical investigations in the OPC

Tests	Area	Specific location	number
X-ray	Thorax		3
	Upper limb		3
	Sacrum		1
	Clavicle		1
CT	Spinal column	Lumbar spine	3
		Cervical spine	1
	Maxillofacial		2
	Lower limb	Hip	2
		Ankle	16
		Foot	4
		Knee	2
		Full leg	1
	Upper limb	Wrist	8
		Hand	1
		Shoulder	3
	Thorax		1
Ultrasound			3
MRI		Knee	3
		Wrist	1
total			59

In a majority of cases the diagnosis mentioned in the ED rapport were distortions (27/56) or contusions (19/56) of the joint or limb. Fifty-seven new diagnoses were made due to further investigation. One patient had two missed fractures (of which one complaint was not registered in the ED files). The most frequent new diagnoses were ankle (13/57), wrist (8/57) and foot (7/57) fractures (Table 4). Avulsion fractures and non-displaced (burst) fractures were most commonly missed. Except for one case with full rotator cuff rupture, no soft tissue lesions were diagnosed.

**Table 4:** Diagnosis

Area	Specific location	Diagnosis	Number	Diagnostic error
Face	Nose	Os nasale fracture	2	
Upper limb	Shoulder	Clavicula fracture	1	
		Rotator cuff rupture	1	
	Elbow	Avulsion fracture	1	
	Wrist	Carpal bone fracture	3	
		Radial metafyse fracture	4	
		Scapholunar ligament rupture	1	
	Hand	Metacarpal fracture	2	
	Humerus	Pathologic humerus fracture	1	1
	Lower arm	Radial burst fracture	1	
		Mid ulna fracture	1	
Lower limb	Hip	Major tubercle fracture	1	
	Knee	Patella avulsion fracture	3	
		Tibia plateau fracture	2	
	Ankle	Talar avulsion fracture	5	
		Tibia fracture	4	
		Fibula fracture	1	
	Foot	Talar dislocation	1	
		Calcaneus avulsion fracture	2	
		Calcaneus fracture	1	1
		Metatarsal fracture	4	
		Tarsal and metatarsal fracture	2	1
	Femur	Femoral condyl fracture	1	
	Tibia/fibula	Distal tibial fracture	1	
Pelvis		Os sacrum	1	
		Pelvic ring fracture	2	1
Spinal column	Cervical spine	C7 fracture	1	
	Thoracic spine	Burst fracture	1	
	Lumbar spine	Burst fracture	2	1
Thorax	Rib	Non displaced rib fracture	3	
	Sternum	Manubrium	1	1
Total			57	6

In only two cases reasonable doubt about the diagnosis, with negative x-rays, was mentioned in patient files and patients were deliberately referred for further investigation. All other patients were referred to the OPC in accordance to hospital protocol and standard of care.

Two soft casts were applied, in 28 patients a soft immobilizing bandage was applied. Twenty three patients had to be referred for specialist advice with new diagnoses having a (significant) impact on treatment varying from cast application to surgical procedure.

DE was suspected in ten cases after record evaluation by the first investigator. In six cases (0.14% of minor trauma patients) DE was confirmed after evaluation by the second investigator. In one specific case (a pathologic arm fracture in a 2 year old boy) DE and negligent care were suspected due to incomplete physical examination and failure to request adequate radiologic investigation. In three cases both radiological investigation and clinical presentation suggested a fracture. In the two remaining cases patient complaints were not pursued during clinical or technical investigation but eventually led to missed fractures.

## Discussion

Emergency physicians have to remain vigilant to prevent and avoid PMD and DE, certainly in case of minor trauma patients, representing a large proportion of ED patients. Several studies have already demonstrated that missed or misdiagnosed fractures are among the most frequently missed diagnoses [1, 2, 8, 15]. In our study we observed a prevalence of 1.39% of missed diagnoses within a six month study period. This is comparable to previous studies with a relative prevalence of 1% [2, 8], however in our study both primary missed diagnoses and DE were included. Using this definition we saw that only one case could be attributed to negligence and DE had a prevalence of 0.14% (6 cases).

Most previous articles concerning missed diagnosis and DE are retrospective in nature. Our study is no different in this aspect. We are aware of the limitations entailed to this type of study.

1. Possible inclusion selection bias due to loss of follow up. With an attendance of 45.6% at the OPC in comparison to the total number of patients seen at the ED a certain number of patients with missed diagnoses were not detected.
2. The retrospective study nature has its limitations. In particular, all information is dependent on the adequacy of Doctors administration and notes. It is not implausible that in several cases history taking and physical examination might have been performed better than perceived in the notes.
3. This study was a single centre study. Possibly our data are not comparable to other EDs due to differences in primary care and follow up.
4. Statistical significant differences in age and time of presentation were not of clinical significance and could be attributed to sample size differences.
5. Our study design did not permit us to investigate all aspects related to DE. Previous work on DE already demonstrated that DE is a multifactorial problem. Considering these limitations we mainly investigated human factors causing diagnostic failure (cognitive and skill-set error) [11].

Despite the relatively low percentage of missed diagnoses, they nevertheless have a negative impact on patients (incorrect or inadequate therapy, prolonged pain, postponed recovery, prolonged work incapability), hospitals (increased workload, revisits, negative publicity, medico-legal liability) and medical services (financial burden).

In comparison to previous studies, we added the concept (and definition) of PMD. Sohn et al. already pointed out that only a minority of missed diagnosis could be attributed to DE and/or negligence [16]. A majority of cases were caused by system errors or could not be attributed to error. By adding the definition of PMD we tried to make a more clear distinction between an unavoidably missed diagnosis and avoidable missed diagnosis in the ED.

We detected 56 patients with in total 57 missed diagnoses. Within this group six out of 56 cases were considered to be the result of DE of which only one case was due to negligence. Based on these data it was not possible to detect a link between the ED system used and the frequency DE or PMD. No patient factors (complaint acuity, influence of drugs/alcohol, neurological state, etc.…) that might contribute to error were noted in patient files, but cannot be entirely excluded. We did reveal some cognitive and skillset errors, as described further on in this discussion. The causes for DE in our study could be categorized into two main groups: failure to perform adequate history taking and/or physical examination and failure to order or correctly interpret technical investigation.

We noticed that in 35.7% of all cases physical examination notes were insufficient. In four out of six DE cases physical examination was insufficient according to trauma history. History taking was insufficient in 30.4% of all cases according to the presenting complaint and/or final diagnosis. Previous studies have already determined that inadequate physical examination and history taking were frequently the cause of DE [2, 12, 17]. Failure to perform adequate physical examination can cause misinterpretation of symptoms or failure to order pertinent technical investigations.

International validated clinical decision rules (Ottowa Ankle and knee rules,…) exist to determine whether there is risk for fracture and consequently a need for X-ray investigations [12–14]. However, uncertainty remains in other major joints and the upper limb due to absence of validated physical examination techniques, although approximately 50% of extremity injuries and fractures occur in the upper limb [12]. To date we adhere to golden standard teaching and carefully note findings in patient files.

In our ED, similarly to previous studies, the mainstay first line technical investigations remains the plain X-ray. In contrast to previous studies however a 24 hour permanence service is provided by the Radiology department, thus reducing the chance of misinterpretation of X-rays [18].

Concerning the typology of new diagnoses, the majority of missed fractures were ankle, wrist and foot fractures. Most fractures were avulsion fractures or non-displaced fractures. Considering previous studies it is unsurprising that these fractures can be missed within the acute phase shortly after trauma. Follow up has to be provided and in case of persistent pain or dysfunction one has to assume the possibility of underlying fracture and second X-ray or a CT scan has to be performed.

We deliberately excluded knee trauma with a trauma mechanism suggesting a soft tissue/tendon lesion, planned follow up and/or investigations. However direct knee trauma, which were considered to be mere contusions, were included. We were able to detect two tibia plateau fractures on CT scan after direct trauma. This type of fractures is known to be an orthopedic pitfall [19]. Due to the anatomical complexity of the knee one must always consider hidden soft tissue and bone trauma and follow up has to be provided.

We were able to detect four spinal and three pelvic fractures during follow up. Sixta et al. [20] demonstrated that up to 25% of all spinal burst fractures were missed on plain X-ray. Although there are no clinical decision rules for thoracic and lumbar spinal trauma, trauma mechanism, age, gender, patient history and clinical symptoms are of importance in determining the need for - and type of - technical investigation. In case of the cervical spine, the Canadian C-spine rules are a reliable and internationally validated method to evaluate the necessity of radiologic investigation [13, 20]. Similarly, up to 30% of pelvic fractures will be missed on plain X-ray [21]. In case of negative x-ray and inability to put full weight on the affected side, one must consider performing additional CT scan in the ED to exclude underlying fractures.

Planned follow up of minor trauma patients who are discharged from the ED with negative investigations should be provided, whether it be at General Practitioners office at Specialists office or at an OPC (as provided in our institution). Ambulatory follow up has several advantages. Firstly, there is a reduction in the number of return visits by dissatisfied patients with persistent complaints to the ED thus reducing workload in the ED. Secondly it gives the opportunity for follow up in case of doubt. Thirdly one can plan secondary investigations, thus reducing workload in the ED rendering better service to our patients. This implies cooperation of patients and the willingness to adhere to planned follow up on the one hand and adequate and full explanations by the emergency physicians on the other hand.

In future studies and follow up we would advise a more extensive inquiry into all aspects leading to diagnostic error in the ED.

## Conclusion

Both primary missed diagnosis and diagnostic error have relatively low prevalence but can have a serious impact on patients, hospitals and medical services. Planned follow up is an important tool to timely detect primary missed diagnosis and prevent diagnostic error after ED admission. Risks for medico legal litigation can be largely prevented by giving adequate information to patients and offering adequate follow up. By organizing teaching moments on screening tools, history taking and adapted technical investigations in function of trauma severity and mechanism we anticipate to reduce diagnostic error in the ED to an absolute minimum (<1%). Further studies should apply a wider (multidimensional) approach to reveal systemic and human factors causing diagnostic error and primary missed diagnosis. Reassessing the frequency of diagnostic error on a timely basis can be used as a key performance indicator in a quality assessment program.

## Abbreviations

DE: Diagnostic error
ED: Emergency Department
OPC: Outpatient Clinic
PMD: Primary missed diagnosis
